# Formwork Technique with Mesh in Elevations of Sinus Floors with Large Perforations of the Schneider Membrane: A Case Pilot

**DOI:** 10.3390/reports7040113

**Published:** 2024-12-12

**Authors:** Erick Rafael Fernández Castellano, Cosimo Galletti, Javier Flores Fraile

**Affiliations:** 1 Department of Surgery, University of Salamanca, 37008 Salamanca, Spain; j.flores@usal.es; 2 School of Medicine and Surgery Kore, University of Enna, 94100 Enna, Italy; cosimo.galletti@unikore.it

**Keywords:** maxillary sinusitis, sinus floor augmentation, titanium mesh

## Abstract

**Background and Clinical Significance**: Currently, maxillary sinus floor elevation is one of the most common procedures used in implantology practice. Despite its predictability, the technique is not without complications, such as graft material dispersion in the sinus cavity, wound dehiscence, hematoma, fenestrations, oroantral fistulas, epistaxis, acute sinusitis, and Schneider membrane perforations. The treatment of the latter can be complex, and depending on its extent, surgery deferral may be necessary, leading to increased patient morbidity. **Case Presentation**: A patient with apical surgery underwent sinus floor elevation with a significant Schneider membrane perforation using a new approach involving titanium mesh, resorbable membrane, and xenograft. This allowed the continuation of surgery, reducing the number of interventions and patient morbidity. **Conclusions:** Despite limitations due to a small sample size, this case report demonstrates that addressing large Schneider membrane perforations and placing implants is effective and predictable using the technology and approach of mesh formwork with titanium.

## 1. Introduction and Clinical Significance

Currently, maxillary sinus floor elevation procedures are widely used in oral surgery and implantology, particularly in cases of insufficient bone height due to posterior sector atrophy and maxillary sinus pneumatization, hindering conventional implant placement [1,2,3]. Age and edentulism cause an increase in maxillary sinus pneumatization, which lowers the maxillary alveolar ridge and leaves the bone too short to support dental implants. Therefore, in order to attain a satisfactory bone volume, the sinus floor needs to be raised [4].

As a less intrusive method, Summers popularized the crestal approach in conjunction with bone transplants and osteotomes [5,6]. In their comparison of sinus elevation utilizing osteotomes with and without grafting, Lai et al. found no discernible variations in implant survival rates between the two groups, with cumulative survival rates for the two groups being 97.18% and 92.13%, respectively [7].

However, the first method described by Tatum comprised a series of incisions that made it possible to reflect a buccal flap, exposing the sinus’s external bony wall. A window was then created to enter the sinus cavity and raise the Schneider membrane in order to introduce a bone graft, which could be autologous bone, bone substitutes, synthetic biomaterials, or a combination of these materials [8].

Graft material dispersion in the sinus cavity, wound dehiscence, hematomas, implant migration into the sinus, fenestrations, oroantral fistulas, epistaxis, bone sequestration, acute sinusitis, and Schneider membrane perforations are among the complications that can occur even though sinus floor elevation is a predictable technique [9,10,11,12,13,14]. With a reported proportion of 35%, Schneider membrane perforation is the most frequent complication, particularly in procedures involving a lateral window (Jensen et al.) [15,16]. In their comprehensive review and meta-analysis, Diaz Olivares et al. found similar results, estimating it to be 30.6% of 1598 procedures [17]. A frequency of 85% of patients with a residual ridge of 3 mm was found by several authors, including Ardekian et al. [18]. This is the cause of the ongoing search for methods to reduce the quantity of the same.

Normally in large perforations the tendency is to close the flap and postpone surgery to reattempt sinus lift after a few months. The purpose of presenting this case is to introduce a new approach to address large perforations or perforations larger than 10 mm, through the use of titanium mesh technology and shuttering, allowing the continuation of surgery and thus reducing the number of interventions and patient morbidity.

## 2. Case Presentation

**Figure 1:**
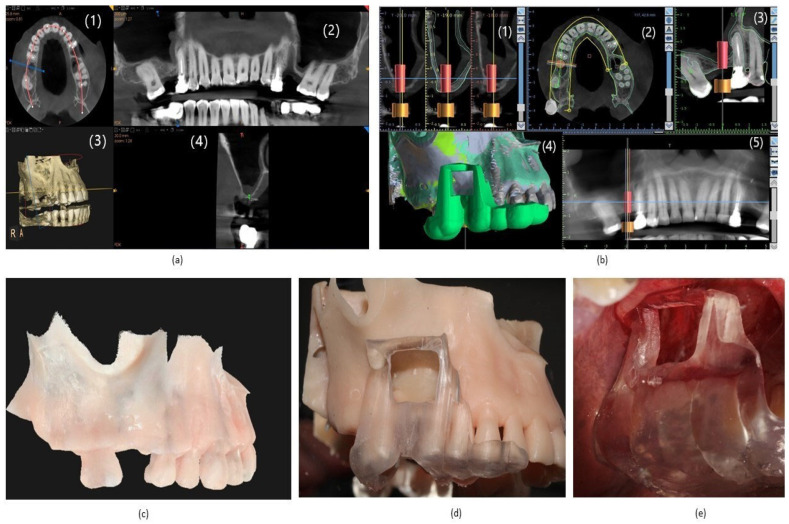
(**a**) Initial planning with CBCT. It shows insufficient height for implant placement. (1) CBCT coronal slice; (2) CBCT panoramic frontal slice; (3) 3D reconstruction; (4) CBCT sagittal slice. (**b**) Planning and design with Blue Sky Bio software for the fabrication of the surgical guides used in the surgeries. (1) Blue Sky Bio sagittal slice; (2) Blue Sky Bio coronal slice; (3) Blue Sky Bio frontal slice; (4) Blue Sky Bio 3D reconstruction; (5) Blue Sky Bio panoramic frontal. (**c**) Stereolithographic model of the patient’s maxillary sinus for surgery simulation. (**d**) Verification of the surgical guide previously designed and printed on the stereolithographic model for the design of the maxillary sinus window. (**e**) Transfer by means of the surgical guide of the stereolithographic model measurements for the creation of the access window.

A 60-year-old female patient, with no significant medical history, visited the clinic to restore the absence of the upper-right first molar with implants. Cone beam computed tomography (CBCT) scans with a 8 × 9 cm field of view were performed using a CS 8100 3D system (Carestream, Rochester, NY, USA) and noting insufficient bone height (see Figure 1). A decision was made to perform maxillary sinus floor elevation surgery with a lateral window approach. Additionally, apical surgery was planned for the symptomatic apical periodontitis of the upper second premolar.

An intraoral scan was performed with a CS 9600 (Carestream, Rochester, NY, USA) and integrated with the CBCT using Blue Sky Bio software (Verison 4.12) for the creation of a surgical guide for both sinus lift surgery and the placement of a prosthetically oriented implant. A stereolithographic model was also printed with dental 3D printing (Ackuretta, Taipei, Taiwan) to check the fit of the surgical guide and simulate both surgeries. The surgical guide previously designed and printed was checked on the stereolithographic model and the opening of the window through the guide was performed.

Once the guide had been prepared and the simulation had been performed, the surgical intervention began after local anesthesia with 1.8 mL of 2% lidocaine and 1:80,000 epinephrine (Xilonibsa; Inibsa, Lliçà de Vall, Spain). A trapezoidal incision accompanied by two vertical incisions in the vestibular mucosa, one distal to the canine and the other mesial to the second molar, was made. A lateral window was then created by transferring the dimensions of the stereolithographic model previously fabricated with the surgical guide (see Figure 1 below).

**Figure 2:**
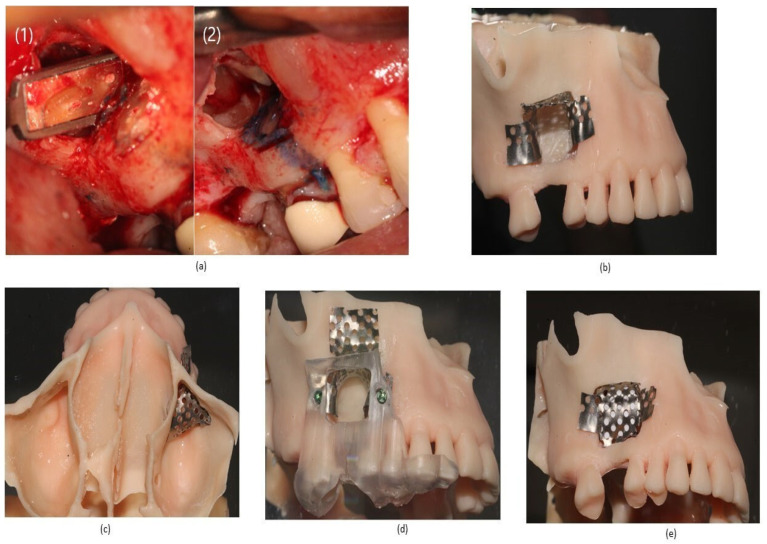
(**a**) Apical surgery: (1) retrograde obturation, (2) note the large perforation of the Schneider membrane. (**b**) Customized titanium mesh formwork design adapted to the stereolithographic model for subsequent transfer to the patient’s mouth. (**c**) Simulation of the formwork inside the sinus in caudal view. (**d**) Surgical guide previously used holding the formwork for its fixation. (**e**) Front view of the formwork with closed window and collagen membrane covering its interior to avoid loss of biomaterial.

When the access window was opened, a large perforation was observed both in the micromirror check in the apical surgery and in the retrofilling. While the apical surgery was being completed, the shaping of the new approach with titanium mesh was performed on the stereolithographic model. Two holes were opened in the surgical guide previously made in the exact places where we wanted to place the screws in order to allow the screw heads to pass through so that the guide would hold the mesh formwork in the exact place of the fixations (see Figure 2 below).

Once adapted to the model, the surgery continued with the novel approach using a 60 × 30 × 0.11 mm titanium mesh designed by the author on the patient (Osteogenos, Torrejón de Ardoz, Spain), which was fixed with 1.2 × 4 mm Global D screws (Brignais, France). The formwork was covered with a layer of Evolution x-fine collagen membrane (Osteobiol, Giaveno, Italy; 30 × 30 mm), which was subsequently filled with Gen-Os biomaterial (Osteobiol, Giaveno, Italy; granulometry 0.25–1 mm/1–2 mm).

After 6 months, a 5 × 5 CBCT was performed before implant placement, revealing bone gain and the absence of sinus pathology.

**Figure 3:**
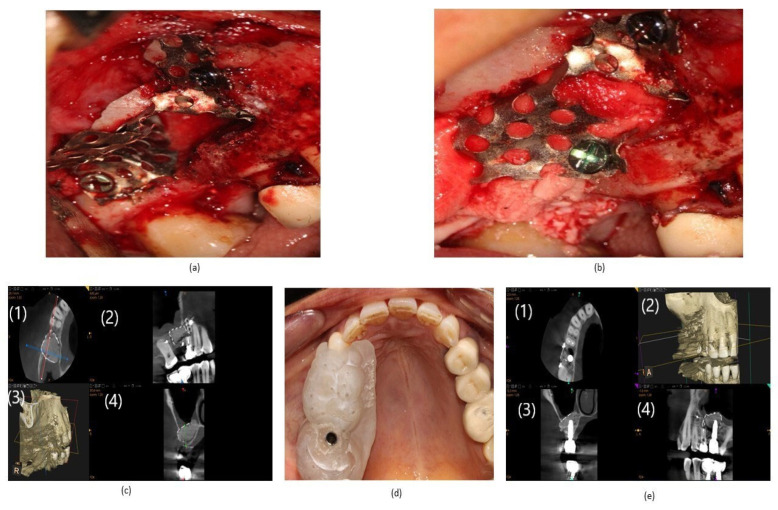
(**a**) Design of the customized titanium mesh formwork fixed inside the patient’s maxillary sinus. (**b**) Formwork with collagen membrane and biomaterial before closing the formwork window. (**c**) The 5 × 5 oblique CBCT slice prior to implant placement, showing the absence of sinus pathology and bone gain. (1) CBCT coronal slice; (2) CBCT frontal slice; (3) 3D reconstruction; (4) CBCT sagittal slice. (**d**) Prosthetically guided implant placement using a previously fabricated surgical guide. (**e**) Two-year follow-up CBCT showing implant continuity and the absence of sinus pathology. (1) CBCT coronal slice; (2) 3D reconstruction; (3) CBCT sagittal slice; (4) CBCT panoramic frontal slice.

The placement of a prosthetically guided 4 × 10 implant (Neobiotech, Seoul, Korea) was carried out using a previously fabricated surgical guide, with a torque of 40 ncm and an ISQ of 72 measured with an Osstell device (W&H, Gothenburg, Sweden). After two years, a follow-up CBCT was performed, revealing the implant’s continuity and the absence of sinus pathology (Figure 3 below).

## 3. Discussion

Preserving the integrity of the Schneider membrane and repairing any perforation is of vital importance for the success of sinus lift [19,20]. Membrane perforations are a relatively frequent intraoperative event in lateral window procedures [21,22] to the point that they are considered the most frequent complication in this type of surgery (7–60%) [16]. Although sinus lifts are fairly frequently performed surgeries, no evidence-based guidelines for perforator closure or clear indications for when to discontinue these procedures have been established [23].

Collagen membrane restoration was the method most frequently employed among the therapies suggested in the literature. Collagen membranes, however, have a number of disadvantages. Testori et al. [24] demonstrated that the use of a collagen membrane in cases of extensive perforations carries the danger of displacement upon implantation of the graft material, resulting in inadequate containment of the material. Hence the importance of the technique proposed in this case to provide support and stability to the collagen membrane.

In 2003, Fugazzotto and Vlassis classified Schneider membrane perforations into three types (I, II, and III) based on their location and provided guidelines for their treatment [25]. According to a systematic review and meta-analysis conducted by Díaz-Olivares et al., for perforations < 5 mm, attempts can be made to fold the membrane, suture it, or use a collagen membrane [17,23,26].

A slow-resorbing collagen membrane is the most suggested treatment for holes between 5 and 10 mm [22,27], allowing the membrane to renew and aiding in the closure of the connection. Resorbable hemostatic agents, or PRFs, are examples of adjuvant treatments.

For perforations > 10 mm, placement of laminar bone with slow-resorbing collagen membranes and implant placement in a subsequent procedure is indicated [28,29]. The use of bone laminae is often challenging to fix, requiring a donor area that involves longer intraoperative time, increased patient morbidity, and a risk of graft dispersion. In the presented case, the formwork technique allowed the continuation of surgery, reducing intraoperative and overall treatment times, and containing the biomaterial in the desired area without the risk of dispersion [30]. The classic publication on the Loma Linda technique in 2003 by Proussaefs and Lozada discusses covering the entire maxillary sinus in large perforations with collagen membrane [31]. However, there is no direct contact with the blood or the bone of the sinus walls, which may result in graft invagination or possible epithelial disease. In the presented formwork technique, as evident in the images, there is direct contact with the medial wall, sinus floor, and blood, promoting graft osteoconduction.

Pikos, in 2008, published an update on the technique for repairing large perforations using collagen membranes that form a kind of formwork through suturing [32]. This allows direct contact of the biomaterial with the patient’s own bone but comes with the drawback of high economic cost and potential graft material dispersion through sutures.

Lopez et al. published in 2024 a new technique called “Sinus Pack” [33], in which they introduced inside the sinus a collagen membrane with the graft material inside rolled up, without fixing it, making it possible to open and detach the particles of biomaterial giving rise to possible sinus pathology with loss of regeneration, as well as a possible invagination of the graft since there is no contact with the walls of the sinus or with the blood that promotes bone formation.

The formwork carried out in our case enables safe working by containing the biomaterial in the desired area without the risk of dispersion. Another significant advantage of the formwork is that in cases of implant failure, the implants would not migrate into the sinus since the mesh’s roof would prevent it, making their easy removal possible. We can also state that it is a less expensive procedure that can be performed completely in the dental office if digital technology such as CBCT, intraoral scanning, and dental 3D printing is available.

In a study, Khalid et al. assessed the use of titanium meshes in maxillary sinus elevations without the use of biomaterials and came to the conclusion that using titanium micromesh as a space-maintaining device following Schneiderian membrane elevation is a reliable method for raising the sinus floor without grafting [34]. Similar results were obtained by Schweikert et al. [35] in their study on the use of titanium mesh in maxillary sinus elevations, where they concluded that thanks to its excellent biocompatibility, numerous patients were treated for two years without complications. In our case, we have also obtained a satisfactory result with a two-year follow-up with the novelty of the use of artificial intelligence and the formwork approach for the satisfactory resolution of large perforations that had not been reported so far.

Regarding the survival of the implants in these types of complications, it is clarified in different publications, in which no differences were found between implants placed in breasts with intact or perforated membranes [10,18,21,36]. Our case with a two-year follow-up confirms this statement. The first findings of this case show favorable outcomes for the novel formwork approach, despite these restrictions and the sample size. Therefore, it has been shown that this approach may be transferred to a clinical setting with more patients and that this technological configuration is generally applicable for upcoming clinical studies.

## 4. Conclusions

Despite limitations due to the small sample size, this case report, which is part of a study, demonstrates that treatment of large Schneider membrane perforations and implant placement is effective and predictable using the digital protocol and titanium mesh shuttering approach described here. Its application can be extended to the field of maxillofacial surgery for reconstruction of atrophic jaws and sinus floor elevations with or without sinus membrane perforation as an alternative to collagen membrane techniques.

## Data Availability

The original data presented in the study are included in the article, further inquiries can be directed to the corresponding author.

## Abbreviations

CBCT	Cone beam computed tomography (CBCT)
CS	Carestream
ISQ	Implant stability quotient
